# Co-Fermentation with *Lactiplantibacillus plantarum* and *Pichia pastoris*: A Novel Approach to Enhance Flavor and Quality of Fermented Tea Beverage

**DOI:** 10.3390/foods14244251

**Published:** 2025-12-10

**Authors:** Jian Li, Yan Chen, Fang Huang, Yan-Tong Liang, Wei-Jian Chen, Yi-Han Cai, Lang-Hong Wang, Yan-Yan Huang

**Affiliations:** Guangdong Provincial Key Laboratory of Intelligent Food Manufacturing, College of Food Science and Engineering, Foshan University, Foshan 528225, China; li_jian@fosu.edu.cn (J.L.); cy0003216@163.com (Y.C.); 16602749429@163.com (F.H.); ytongleung@163.com (Y.-T.L.); mikey_587@163.com (W.-J.C.); 13502631406@163.com (Y.-H.C.); wlhong@fosu.edu.cn (L.-H.W.)

**Keywords:** fermented tea beverage, *Lactiplantibacillus plantarum*, *Pichia pastoris*, microbial diversity, volatile compounds

## Abstract

Fermented tea beverage (FTB) has garnered significant attention owing to its unique combination of tea and wine flavors and its potential health benefits. This study investigates FTB co-fermented using different inoculum sizes of *L. plantarum* HYY-S10 and *P. pastoris*, evaluating physicochemical properties during the fermentation process. The final FTB products were comprehensively evaluated for their antioxidant activity, organic acid content, sensory characteristics, volatile flavor compounds, and microbial diversity. Compared with natural fermentation, the 1:1 mixed fermentation of these two microorganisms enhanced the antioxidant capacity and organic acid content of FTB. Furthermore, sensory evaluation revealed higher overall acceptability. Analysis of volatile compounds demonstrated an increase in the production of alcohols, esters, and ketones, leading to enhanced malty, fruity, and creamy aromas in FTB. Among these compounds, 3-methyl-1-butanol, phenylethyl alcohol, 1,2-propanediol, and 3-hydroxy-2-butanone play pivotal roles in shaping the flavor profile. High-throughput sequencing analysis identified *Lactobacillus* and *Weizmannia* as dominant bacteria, while *Pichia* or *Issatchenkia* was found to be dominant fungi. This study provides a theoretical foundation for the production of FTB through mixed fermentation with *L. plantarum* HYY-S10 and *P. pastoris* while contributing to the practical application of FTB production through mixed fermentation techniques. Collectively, our findings demonstrate that the 1:1 co-fermentation of *L. plantarum* HYY-S10 and *P. pastoris* is a promising strategy for developing novel fermented tea beverages with enhanced functional properties and complex, desirable flavors, offering valuable insights for the industrial production of specialty FTBs.

## 1. Introduction

Tea, a globally popular non-alcoholic drink, is produced from fresh leaves and buds of *Camellia sinensis* (L.) O. Kuntze [1]. The growing preference for convenient tea products has rendered conventional brewing methods insufficient to address contemporary market demands, particularly among younger demographics, thereby stimulating innovation in fermented tea beverages (FTB). Recent years have witnessed significant advancements in tea-based product development, encompassing various innovative formulations such as liquid tea beverage, instant tea powder, tea concentrate, kombucha, tea wine and other fermented tea beverages [2]. Research has demonstrated that FTB provides various health benefits, including antioxidant properties, hypoglycemic effects, diuretic action, fatigue relief, nerve protection, alleviation of gastrointestinal discomfort, and immune enhancement [3,4,5]. As consumer purchasing power increases, organoleptic properties and health benefits of FTB have gained greater prominence in product development.

Microbial co-fermentation has emerged as a pivotal processing technology for enhancing both the organoleptic properties and functional value of FTB [6]. Recent studies demonstrate that synergistic interactions between lactic acid bacteria (LAB) and yeast significantly improve flavor complexity (e.g., increased fruity esters and floral alcohols) while boosting antioxidant capacity and organic acid content, as evidenced in kombucha and other fermented beverages [7]. *Pichia pastoris,* a representative non-*Saccharomyces* yeast, transforms reducing sugars into ethanol and various metabolic by-products during alcoholic fermentation, making it an excellent choice for producing complex flavors and low-bodied wines [8]. Recent years have witnessed growing scientific and industrial attention toward utilizing Lactic acid bacteria (LAB) in FTB production systems. *L. plantarum*, known for its probiotic properties, not only enhances the flavor and quality of fermented foods but also offers potential health benefits [9,10]. Zhao et al. demonstrated that LAB-fermented tea beverages exhibited significantly enhanced DPPH radical scavenging activity and cellular antioxidant capacity compared to conventional tea products [11]. Ping et al. demonstrated that inoculation with *Lactiplantibacillus plantarum* significantly reduced the fermentation duration while simultaneously enhancing the sensory attributes of fermented tea products [12]. Jiang et al. found that *L. plantarum* inoculation increased total flavonoids while reducing acidity and improving sensory quality in prune wine beverage [13]. These studies consistently found that adding LAB to the fermentation process resulted in shorter fermentation times, enhanced antioxidant properties, and improved flavor and organoleptic characteristics.

To date, limited research has been conducted on the production of FTB. While some studies have explored LAB-yeast co-fermentation, the specific synergistic combination of *P. pastoris* and *L. plantarum* represents a significant and unexplored frontier, distinguishing this study from previous work. While *P. pastoris* is renowned as a microbial host for recombinant protein production in molecular biology, its potential in beverage fermentation remains relatively underexplored, particularly in co-fermentation systems. Unlike conventional brewing yeasts (e.g., *Saccharomyces cerevisiae*), which often dominate fermentation and produce strong ethanol-driven profiles, *P. pastoris* typically yields less ethanol and a distinct spectrum of metabolites, making it a promising candidate for developing low-alcohol beverages with unique flavor complexities. This fundamental difference underpins our rationale for selecting *P. pastoris* Mbpk-21, to investigate its underutilized potential and possible synergistic effects with LAB in shaping the organoleptic and functional properties of FTB. Particularly, the strain-specific interactions between *P. pastoris* Mbpk-21 and *L. plantarum* HYY-S10 in the tea matrix warrant systematic investigation. This study investigates FTB co-fermented using different inoculum size of *L. plantarum* HYY-S10 and *P. pastoris* Mbpk-21, evaluating physicochemical properties during the fermentation process. The final products were comprehensively evaluated for their antioxidant activity, organic acid content, volatile flavor compounds, microbial diversity, and sensory characteristics, with further exploration of the intercorrelations among these key parameters. The findings from this research provide a theoretical foundation for producing fermented tea beverages through co-fermentation with yeast and LAB while contributing to the practical application of fermented tea beverages production through co-fermentation techniques.

## 2. Materials and Methods

### 2.1. Materials and Reagents

*Lactiplantibacillus plantarum* HYY-S10 (LP) (CGMCC No. 62784) was originally isolated from de’ang sour tea, while *Pichia pastoris* Mbpk-21 (PP) (GDMCC NO. 62253) was derived from mulberry fruit wine. Both strains were preserved at the Guangdong Microbial Culture Collection Center (Guangdong, China), where they underwent freeze-drying and storage in a −80 °C freezer.

Glutinous rice flour and green tea leaves were obtained from local supermarkets in Foshan, China. Mixed acid standards, methanol, acetonitrile, and ethanol (high-performance liquid chromatography grade) were acquired from Honeywell (Marris Township, NJ, USA). 3,5-dinitrosalicylic acid (DNS), 1,1-diphenyl-2-picrylhydrazyl (DPPH), and 2,2′-azinobis-3-ethylbenzthiazoline-6-sulfonic acid (ABTS) were procured from Fuzhou Phygene Biotechnology Co. (Fuzhou, China). The MRS medium, Bengal red medium, and agar were sourced from Guangdong Huankai Biotechnology Co. (Guangzhou, China). Additional analytical grade chemicals, including sodium chloride, sodium hydroxide, PBS, phenolphthalein indicator, and anhydrous ethanol, were obtained from Macklin Biochemical Co., Ltd. (Beijing, China).

### 2.2. Fermented Tea Beverage Preparation

A mixture was formulated by combining glutinous rice flour with water at a 1:8 ratio, which was then subjected to a 95 °C water bath for half an hour. Following this, amylase (3.28 g, 1000 U/g) was incorporated, and the solution was maintained at 80 °C for 45 min. Upon cooling to ambient temperature, glucoamylase (0.04 g, 1000 U/g) was added, and the mixture was allowed to react at 60 °C for 210 min. The reaction mixture was then exposed to iodine solution and heated again at 95 °C for 30 min, with constant agitation until the disappearance of the blue coloration indicated the completion of the reaction. The resulting saccharified liquid was filtered through a quadruple layer of 100 mesh gauze. Aliquots of the filtrate were transferred to conical flasks and sterilized at 121 °C for 20 min. Finally, green tea leaves were introduced into the solution at a concentration of 2% (mg/mL). To investigate the effect of different inoculum amounts on FTB quality during fermentation, varying ratios of LP and PP were added in a sterile environment. The selected ratios (100:1, 10:1, 1:1, 1:10, and 1:100) were designed to create a gradient that would span from LP-dominant to PP-dominant consortiums, thereby enabling a comprehensive analysis of how the shift in microbial predominance influences the fermentation dynamics and final product characteristics. The natural fermentation group was designated as CK. PP was fixed at a concentration of 6 log CFU/mL, while LP was inoculated at five concentrations (0, 4, 5, 6, 7, and 8 log CFU/mL). These combinations were represented as PP, LPL1, LPL2, LPM, LPH1, and LPH2. Additionally, an inoculation concentration of 6 log CFU/mL of pure LP fermentation was established as the LP group (Table 1). All samples were fermented at 28 °C in a closed environment for 10 days [14].

At designated intervals (0, 2, 4, 6, 8, and 10 days), the samples underwent analysis for viable cell counts, pH, titratable acidity, total esters, and reducing sugar content. Upon completion of the fermentation process, all samples were preserved at −80 °C for subsequent evaluation of alcohol content, antioxidant properties, volatile compounds, microbial diversity, and sensory characteristics.

### 2.3. Determination of Dynamic Changes in Physicochemical Properties

#### 2.3.1. Determination of Viable Count

The viable count of microorganisms was determined in accordance with a previous study conducted by Huang et al. [15]. Each homogeneous FTB sample (100 μL) was diluted using a sterile saline solution (0.9%) with dilution factors ranging from 10^6^ to 10^9^. LAB colonies were enumerated after incubation for 48 h on MRS agar plates at 37 °C, while yeast colonies were counted following incubation for 3 to 5 days on Bengal red agar plates at 28 °C.

#### 2.3.2. Determination of pH, Reducing Sugars, Titratable Acidity, and Total Esters

The FTB samples underwent filtration, and their pH values were measured using a pH meter (PHS-3E, Shanghai, China). Reducing sugar content in the FTB was determined through a colorimetric method employing 3,5-dinitrosalicylic acid (DNS), as outlined by Zhang et al. [16]. Titratable acidity was quantified via potentiometric titration (ZDJ-3A, Shanghai, China) following the methodology established by Han et al. [17], with results expressed as lactic acid content (g/L). Total ester content was assessed using automatic potentiometric titration (ZDJ-3A, Shanghai, China), in accordance with the method described by Ouyang et al. [18], with results expressed as ethyl acetate content (g/L).

### 2.4. Determination of Alcohol Content

Transfer 100 mL of the fermented FTB sample to the distillation flask. The samples were distilled at 78 °C using a laboratory distillation apparatus (the condenser temperature was maintained at 15 °C and a circulating coolant was used). The first 10 mL of the distillate was discarded to remove volatile impurities, and the subsequent 80 mL was collected in a clean, pre-cooled grading cylinder. The sample was placed in a water bath maintained at 20 °C. An alcohol meter (DA-130N, Kyoto Electronics Manufacturing Co., Ltd., Shanghai, China) was vertically inserted into the FTB. The alcohol content (% vol) was recorded once the liquid level (% vol) stabilized.

### 2.5. Determination of Antioxidant Properties

The antioxidant properties of FTB were evaluated using the method described by [19]. For the DPPH assay, samples (100 μL) were reacted with 0.1 mM DPPH solution in 96-well plates at 25 °C for 30 min in darkness, with absorbance measured at 517 nm using a microplate reader (EPOCH2, BioTek Instruments, Inc., Winooski, VT, USA), including appropriate controls (blank: methanol + sample; negative: DPPH + methanol; positive: vitamin C calibration curve). The ABTS assay involved reacting 7 mM ABTS with 2.45 mM K_2_S_2_O_8_ for 16 h to generate the radical cation, which was then diluted to OD_734nm_ = 0.70 ± 0.02 with PBS; samples (10 μL) were reacted with 190 μL ABTS solution for 6 min before measurement.

### 2.6. Determination of Organic Acids

The organic acid composition of the FTB samples was analyzed via an HPLC instrument (LC1200, Agilent Technologies, Santa Clara, CA, USA) equipped with an Art Chrome Organic Acid H^+^ column (8 μm 300 mm × 7.8 mm, Starchrom, Changsha, China). A 20 μL injection volume was used for the analysis, with 5 mmol/L sulfuric acid solution as the mobile phase at a flow rate of 0.5 mL/min. Detection was performed using an ultraviolet detector at 210 nm and 55 °C. The organic acid content in Poria samples was quantified via comparison with corresponding standards.

### 2.7. Sensory Evaluation

The organoleptic quality of the FTB e samples was evaluated using a 25-point scale, with scores ranging from 1 to 25 (1 indicating strong dislike and 25 indicating strong preference). A sensory evaluation team comprising 30 trained experts in the field conducted a comprehensive assessment of the FTB’s appearance, aroma, taste, and style. Prior to formal evaluation, all samples were randomly coded in the preparation area before being presented to the panelists. Prior to participation, the researchers provided informed consent. The study received approval from the Ethics Committee of the Faculty of Food Science and Engineering, Foshan University (SYXK 2020-0235).

### 2.8. Determination of Volatile Compounds Using Gas Chromatography–Mass Spectrometry (GC-MS)

Volatile compounds in the eight FTB samples were analyzed via headspace solid-phase micro-extraction (57330-U HS-SPME, Supelco, Bellefonte, PA, USA) coupled with gas chromatography–mass spectrometry (6890N-5973 GC-MS, Agilent Technologies, Santa Clara, CA, USA). The spectrometer was equipped with an HP-INNOWax capillary column (60 m × 0.25 mm I.D., 0.25 μm df; Agilent Technologies) and the SPME device with fibers (DVB/CAR/PDMS needle, Supelco, Bellefonte, PA, USA). For the analysis of the eight FTB samples, 5.0 mL aliquots were transferred into 20 mL headspace vials and incubated at 80 °C for 30 min to facilitate the adsorption of volatile compounds onto the SPME fiber. The GC conditions were as follows: The temperature program started at 40 °C with a hold time of 5 min, followed by ramping at a rate of 5 °C/min until reaching a final temperature of 250 °C, which was maintained for an additional 10 min. Qualitative identification was performed by comparing mass spectra with the NIST library (match similarity >80%) and authentic standards when available. For quantification, relative abundances (%) were calculated as the peak area of each compound normalized to the total peak area. Finally, we identified several volatile compounds with the highest relative abundances as the key flavor determinants [20].

### 2.9. Determination of Microbial Diversity

Genomic DNA was extracted from fermented tea beverage (FTB) samples using the DNeasy PowerSoil Pro Kit (Qiagen, Hilden, Germany), with purity and concentration verified by 1% agarose gel electrophoresis and Qubit fluorometric quantification (Thermo Fisher, Waltham, MA, USA). Bacterial 16S rRNA gene (V3-V4 region) and fungal ITS1 were amplified using primer pairs 341F/806R and ITS5F/ITS2R, respectively, with Q5 High-Fidelity DNA Polymerase (NEB) under optimized PCR conditions (25 cycles, 55 °C annealing). Amplicons were purified (AMPure XP beads), pooled in equimolar ratios, and sequenced on the Illumina NovaSeq 6000 platform (2 × 250 bp paired-end; Micromax Technology, Shenzhen, China). Raw reads were quality-filtered (Phred score ≥ 20), clustered into operational taxonomic units (OTUs) at 97% similarity, and taxonomically classified against the Greengenes2 (for 16S) and UNITE v9.0 (for ITS) databases with a 0.7 confidence threshold. Negative controls and technical replicates were included throughout the process to ensure data reliability, and sequence data were deposited in the NCBI SRA (BioProject: PRJNA1293861) [21,22].

### 2.10. Data Analysis

Statistical comparisons between samples were conducted via analysis of variance followed by Tukey’s test (*p* < 0.05). For multifactor comparisons, principal component analysis (PCA) and heat map visualization in conjunction with hierarchical cluster analysis based on Pearson’s correlation were performed using SPSS 27.0 (SPSS Inc., Chicago, IL, USA). All experimental data were obtained from three independent replicates for each sample to ensure reliability.

## 3. Results and Discussion

### 3.1. Dynamic Changes Analysis of Fermentation Process

#### 3.1.1. Changes in Viable Counts During Fermentation Process

**Figure 1:**
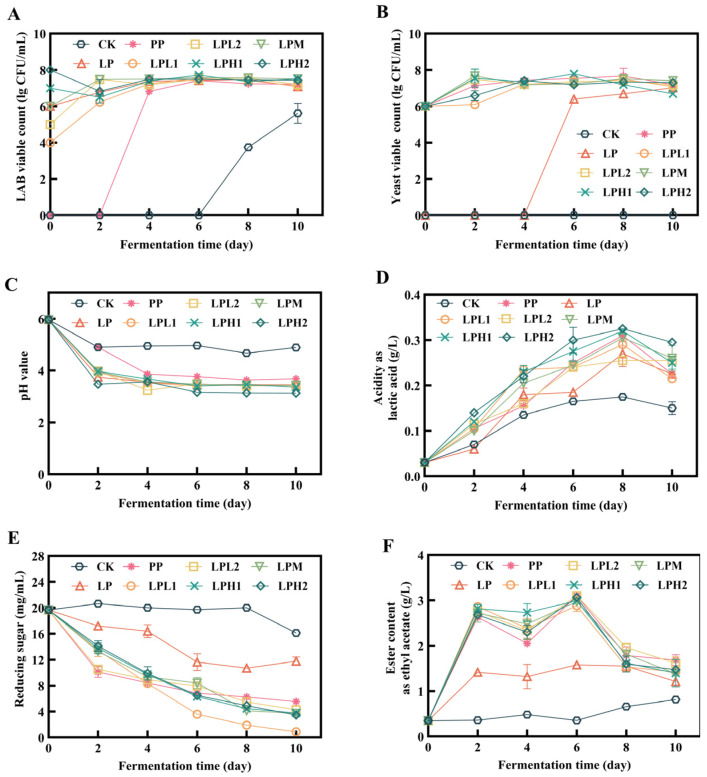
The changes in physical and chemical properties during fermentation. Viable counts of LAB (**A**), viable counts of yeast (**B**), pH (**C**), titratable acidity (**D**), reducing sugars content (**E**), and ester content (**F**). CK: natural fermentation; LP: *L. plantarum* HYY-S10 monoculture; PP: *P. pastoris* monoculture; LPH2, LPH1, LPM, LPL1, LPL2: co-culture groups with *L. plantarum* HYY-S10 to *P. pastoris* inoculation ratios of 100:1, 10:1, 1:1, 1:10, and 1:100, respectively.

For probiotics to be effective, their count in the fermented product must exceed 7 log CFU/mL [23]. As depicted in Figure 1A, with the exception of the CK, LP, and PP groups, all co-fermentation groups demonstrated sustained lactic acid bacteria (LAB) viability within the optimal range of 7–8 log CFU/mL throughout the fermentation process. This viability threshold was similarly maintained for yeast cells across experimental groups (Figure 1B). Notably, the CK group, despite receiving no microbial inoculation, exhibited detectable viable cell counts, likely attributable to indigenous microorganisms naturally present in the tea leaves. These findings collectively demonstrate that the FTB substrate not only provides sufficient nutritional support to sustain robust growth of both *L. plantarum* and *P. pastoris* throughout the fermentation process, but also yields a final product that fully complies with established microbiological standards for probiotic-containing fermented beverages.

#### 3.1.2. Changes in pH and Titratable Acidity During Fermentation Process

All samples exhibited a decreasing trend in pH values, reaching equilibrium after two days of fermentation (Figure 1C). With the exception of the CK group, all experimental groups ultimately achieved pH values below 4.0, with the LPH1 and LPH2 groups demonstrating the most pronounced rate of pH decline. Conversely, titratable acidity (TA) showed an inverse trend (Figure 1D). On the eighth day of fermentation, the LPH2 group reached peak acidity (0.32 g/L), while the CK group maintained the lowest level (0.17 g/L). These results indicate that inoculation with higher doses of *L. plantarum* HYY-S10 during tea beverage fermentation can effectively enhance its acidity. This increase is primarily attributed to the microbial production of organic acids, notably lactic acid from *L. plantarum* metabolism and acetic acid from the synergistic activity with yeast. Low pH and high TA can inhibit the growth of harmful microorganisms and improve the quality of fermented food [24].

#### 3.1.3. Changes in Total Esters and Reducing Sugars During Fermentation Process

Microorganisms predominantly utilize reducing sugars as carbon sources to generate a diverse array of flavor metabolites, including alcohols, organic acids, and esters. The concentration of reducing sugars also serves as a critical indicator for monitoring fermentation progression [25]. Throughout the fermentation process, all samples demonstrated a consistent decline in reducing sugar content, with the exception of the CK group (Figure 1E). By fermentation day 10, the LPL2 group exhibited the most substantial reduction in sugar content, reaching a minimum concentration of 0.56 mg/mL, while the CK group maintained the highest residual sugar level at 17.40 mg/mL. These results demonstrate that the symbiotic fermentation system involving *L. plantarum* HYY-S10 and *P. pastoris* facilitates more efficient catabolism of reducing sugars into organic acids and other flavor-active compounds, thereby improving the overall fermentation efficiency and flavor profile development in FTB production. The rapid decline in reducing sugars provides ample precursors (primarily organic acids and alcohols) for the subsequent synthesis of esters.

Esters serve as the predominant aromatic compounds that determine the characteristic flavor profile of fermented food. During fermentation, the total ester content in FTB samples exhibited an initial increase before subsequently declining (Figure 1F). The initial increase corresponds to the active esterification of the accumulated acids and alcohols derived from sugar metabolism. Notably, co-fermentation systems consistently demonstrated significantly higher ester accumulation compared to the CK control group. On day 6 of fermentation, the CK group showed minimal ester production (0.35 g/L), while the LPL1 and LPL2 co-culture groups achieved peak concentrations of 2.98 g/L and 3.00 g/L, respectively. The observed 8.5-fold increase in ester production under optimal co-culture conditions suggests synergistic interactions between microbial species, potentially involving substrate competition and metabolic cross-feeding mechanisms that promote flavor compound biosynthesis.

### 3.2. Analysis of Changes in Antioxidant Properties

**Figure 2:**
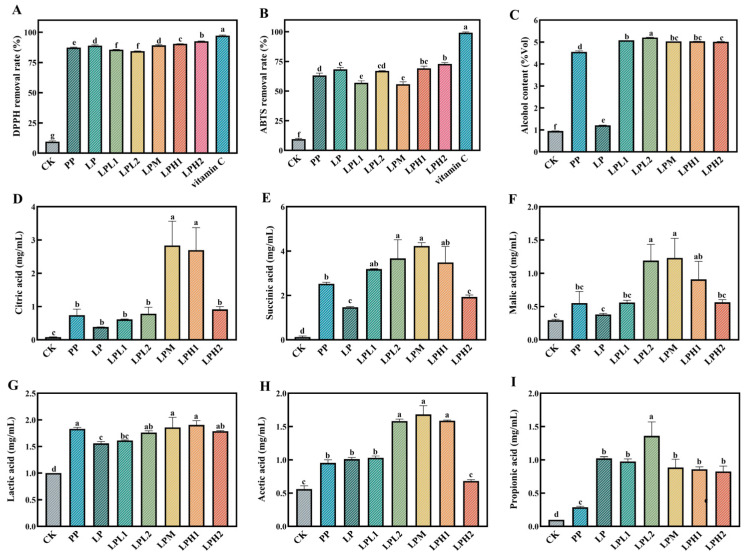
Analysis of the antioxidant capacity, alcohol and organic acid content of the final product. DPPH removal rate (**A**), ABTS removal rate (**B**), alcohol content (**C**), citric acid (**D**), succinic acid (**E**), malic acid (**F**), lactic acid (**G**), acetic acid (**H**), propionic acid (**I**). CK: natural fermentation; LP: *L. plantarum* HYY-S10 monoculture; PP: *P. pastoris* monoculture; LPH2, LPH1, LPM, LPL1, LPL2: co-culture groups with *L. plantarum* HYY-S10 to *P. pastoris* inoculation ratios of 100:1, 10:1, 1:1, 1:10, and 1:100, respectively. Lowercase letters indicate significant differences among groups.

The symbiotic fermentation system employing *L. plantarum* and *P. pastoris* demonstrated superior antioxidant potential in FTB production compared to natural fermentation (Figure 2A,B). Quantitative analysis revealed the LPH2 group exhibited optimal antioxidant performance, attaining exceptional scavenging activities of 92.57% (DPPH-) and 72.89% (ABTS+), representing significant improvements over CK group. This enhancement in antioxidant activity can be attributed to the synergistic effects of metabolites produced by *L. plantarum* HYY-S10 during fermentation, such as organic acids, bacteriocins, and bioactive peptides, as well as the inherent phenolic compounds present in tea, including catechins and flavonoids [5]. These compounds collectively contribute to the stabilization of free radicals and the overall improvement of the FTB’s oxidative stability. Furthermore, the interaction between *L. plantarum* HYY-S10 and *P. pastoris* likely facilitated the bioconversion of phenolic precursors into more bioactive forms, thereby amplifying the antioxidant potential of the final product.

### 3.3. Analysis of Changes in Alcohol Content

As shown in Figure 2C, the alcohol content analysis revealed significant differences among the eight FTB samples. Notably, the co-fermentation system employing both *L. plantarum* HYY-S10 and *P. pastoris* consistently produced FTB with alcohol contents exceeding 5.00 vol%, resulting in a beverage with a moderate alcohol level that was distinctly higher than that achieved through either natural fermentation or single-strain fermentation. This marked improvement in ethanol yield likely results from the metabolic synergy between the two microbial species, where their complementary enzymatic activities may have enhanced the complete utilization of available fermentable substrates while optimizing the glycolytic pathway for ethanol biosynthesis [7]. The findings suggest that this microbial consortium establishes a more efficient fermentation microenvironment that maximizes the bioconversion of carbohydrates to ethanol.

### 3.4. Analysis of Changes in Organic Acid

As illustrated in Figure 2D–I, a comprehensive analysis of the eight experimental groups at the fermentation endpoint identified six organic acids: citric, succinic, malic, lactic, acetic, and propionic. These results align with prior research on tea wine chemistry [26]. Quantitative analysis revealed in total organic acid content among treatment groups, ranging from 4.97 mg/mL (CK group) to 14.13 mg/mL (LPM group). The organic acid profile of fermented tea beverage (FTB) samples was characterized by five predominant constituents: citric, malic, succinic, lactic, and acetic acids. The LPM treatment group contained significantly elevated concentrations (*p* < 0.05) of these key acids compared to other experimental groups. The biochemical origin of these acids reflects distinct metabolic pathways: citric and succinic acids are direct products of LAB activity in the tricarboxylic acid (TCA) cycle, while acetic and isobutyric acids derive from heterofermentative LAB metabolism [27]. These organic acids serve dual functions—they directly contribute to the tart, complex flavor profile of FTB while also serving as precursors for ester formation. Subsequent esterification reactions with ethanol yield important flavor-active compounds such as ethyl acetate (fruity notes) and isobutyl acetate (banana-like aroma), which significantly enhance the organoleptic quality of the final product [7].

### 3.5. Analysis of the Sensory Evaluation

**Figure 3:**
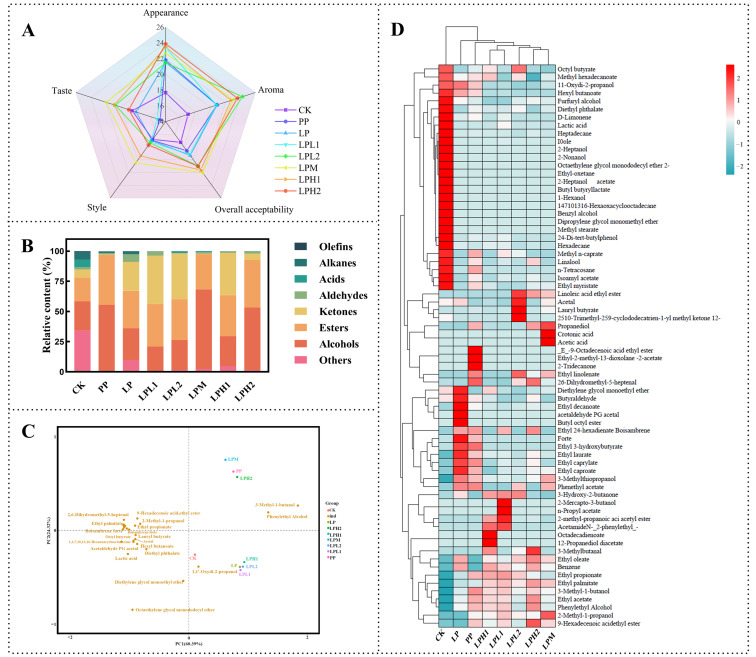
Analysis of sensory evaluation and volatile compounds. Sensory evaluation radar chart (**A**), category stacking chart (**B**), principal component (PC) biplot of relative content of selected major volatile compounds (**C**), and heat map and clustering of the selected major volatile compounds (**D**). CK: natural fermentation; LP: *L. plantarum* HYY-S10 monoculture; PP: *P. pastoris* monoculture; LPH2, LPH1, LPM, LPL1, LPL2: co-culture groups with *L. plantarum* HYY-S10 to *P. pastoris* inoculation ratios of 100:1, 10:1, 1:1, 1:10, and 1:100, respectively.

Comprehensive sensory evaluation was conducted on all FTB samples, with independent assessments of five key quality attributes: appearance, aroma, style, taste, and overall acceptability (Figure 3A). The LPM group achieved the highest composite sensory scores, demonstrating statistical improvements of 11.01% to 20.36% over the CK, LP, and PC groups. The combined fermentation of *L. plantarum* HYY-S10 and *P. pastoris* primarily enhanced the aroma (characterized by pronounced fruity and floral notes) and appearance (notably brighter color and clearer transparency) of the FTB (Figure 3A). This enhancement is attributed to the organic acids, esters, and other flavor compounds produced by *L. plantarum* HYY-S10 during FTB fermentation [27]. Additionally, the amino acids and alcohol generated by *P. pastoris* formed esters with the organic acids during the fermentation process. These compounds collectively contributed to the improved flavor profile of the FTB, marked by reduced astringency [28].

### 3.6. Analysis of the Volatile Compounds

To comprehensively characterize the aromatic profiles of the FTB samples, we performed detailed volatile compound analysis using HS-SPME-GC-MS. A total of 145 major volatile compounds were identified, comprising alcohols (23), esters (53), aldehydes (20), ketones (9), acids (5), alkanes (13), olefins (7), and other substances (14) (Table S1). Notably, the flavor profile was dominated by alcohol, ester, and ketone compounds, which collectively represented more than 90% of the total volatile content (Figure 3B). This compositional pattern is consistent with previous findings by Xu et al. in their characterization of dark tea wine volatiles, confirming the characteristic flavor chemistry of tea-based fermented beverages [29]. Principal Component Analysis (PCA) of the volatile profiles yielded particularly insightful results, with the first two principal components (PC1 and PC2) explaining 84.71% of the total variance (60.39% and 24.32%, respectively) (Figure 3C). The PP, LPM, LPH2, and CK, LP, LPH1, LPL1, LPL2 groups clearly made a distinction among each other by PC2 (24.32% of variance). PC2 was correlated negatively with 11 volatile substances at higher concentrations among these FTB samples. The flavor score of PP, LPM, and LPH2 groups were positively associated with 2 volatile substances (3-Methyl-1-butanol and phenylethyl alcohol). These findings were consistent with the sensory panel’s higher ratings for “fruity” and “floral” attributes in these groups, suggesting these volatiles serve as important flavor determinants in FTB. Furthermore, hierarchical clustering analysis (HCA) was performed to visualize the differential abundance patterns of volatile flavor compounds among the various FTB fermentation groups (Figure 3D).

**Alcohols:** Alcohols serve as critical aromatic and structural components in FTB, with their diversity and concentration directly shaping the beverage’s organoleptic profile. As illustrated in Figure 3B, the LPM achieved the highest total alcohol content (65.77% of total volatile compounds), significantly outperforming the control group (CK, 24.21%). This pronounced disparity likely stems from synergistic microbial interactions under anaerobic conditions, where *L. plantarum* HYY-S10 enhances substrate availability for *P. pastoris*, thereby optimizing yeast-driven alcohol biosynthesis via the Ehrlich pathway. In this pathway, amino acids undergo transamination to form α-keto acids, which are subsequently decarboxylated and reduced by alcohol dehydrogenase (ADH) to yield higher alcohols [30]. Table S1 analysis identified seven characteristic alcohols across all FTB samples (1,2-propanediol, phenylethanol, linalool, 2-methyl-1-propanol, 3-methyl-1-butanol, 1,1′-oxydi-2-propanol, and 3-methylmercaptopropanol), with 3-methyl-1-butanol, phenylethyl alcohol, and 1,2-propanediol demonstrating the most significant aromatic contributions. The malty and fruity notes of 3-methyl-1-butanol, combined with the distinct floral and honey-like aromas of phenylethyl alcohol, synergistically enhanced the flavor complexity, while 1,2-propanediol modulated mouthfeel through subtle sweetness and viscosity [31]. The superior alcohol profile of the LPM group likely stems from its optimized microbial ratio, which facilitates nitrogen metabolism for amino acid precursor availability, maintains redox equilibrium to sustain alcohol dehydrogenase activity, and promotes esterification between alcohols and organic acids for enriched secondary flavor development.

**Esters:** The characteristic fruity and floral aromas of tea wine predominantly originate from ester compounds synthesized through enzymatic esterification between carboxylic acids and alcohols, a critical biochemical process in flavor development [27]. GC-MS analysis identified 53 distinct esters across the eight FTB variants, with co-fermented samples (*L. plantarum* HYY-S10 + *P. pastoris*) exhibiting a significantly higher relative ester content (29.36–41.42%) compared to naturally fermented controls (CK, 19.62%) (Table S1). This 1.5- to 2.1-fold enhancement in ester production highlights the metabolic synergy between LAB and yeast, where *L. plantarum* HYY-S10 supplies abundant organic acids (e.g., acetic, lactic) while *P. pastoris* generates ethanol and higher alcohols, creating optimal conditions for ester biosynthesis. These results indicated that the co-fermentation of FTB with LAB and yeast not only produced a greater quantity of ester compounds compared with naturally fermented FTB but also enhanced the overall aroma profile of FTB. Among the esters identified, isobutyl acetate, ethyl acetate, and isoamyl acetate were present in high relative content (Table S1) and were formed by the esterification of ethanol and acetic acid. These compounds play a significant role in generating a fruity aroma, with isoamyl acetate contributing to a sweet banana-like fragrance.

**Ketones:** The ketone profile of FTB is shaped through multiple biochemical pathways, including oxidative transformations of polyphenols, thermal degradation of precursors, amino acid catabolism, and specialized microbial metabolism [32]. GC-MS analysis revealed 3-hydroxy-2-butanone as the dominant ketone, accounting for 38.11% and 38.83% of total volatile compounds in the LPL1 and LPL2 groups, respectively (Table S1). This key flavor metabolite, known for its characteristic creamy/buttery notes. These findings align with recent studies demonstrating acetoin’s crucial role in developing the signature flavor profile of fermented tea products [33]. This enhanced ketone production may be attributed to tea-specific substrates serving as preferential precursors for microbial biotransformation.

The organic acid composition in tea wine predominantly stems from lactic acid bacteria (LAB) metabolic activity, though their final concentrations appear limited due to extensive esterification processes with alcohols during fermentation [27]. The analytical results identified merely five organic acids present at relatively low concentrations (Table S1), a phenomenon attributable to their rapid conversion into flavor-active esters. This metabolic transformation similarly accounts for the trace levels of aldehydes, alkanes, and olefins detected (Table S1), as these intermediate compounds are efficiently metabolized within the dynamic fermentation system. The co-fermentation approach employing LAB and yeast consortiums exhibited superior metabolic capability compared to natural fermentation, particularly in generating elevated quantities of key flavor compounds including fusel alcohols, diverse esters, and ketones. This enhancement can be attributed to the well-established metabolic synergy in such systems: yeast primarily generates ethanol and fusel alcohols, which subsequently serve as precursors for ester synthesis [34]. LAB, in turn, produces organic acids that can also act as substrates for esterification [35]. The co-culture environment thus establishes a more efficient and diverse metabolic network for the biosynthesis of flavor compounds than the undefined and often less efficient microbial community in natural fermentation [36]. This enhanced biosynthesis directly contributes to the development of a more complex and desirable aromatic profile in FTB, characterized by pronounced malty, fruity, and creamy notes.

### 3.7. Analysis of Microbial Diversity and Composition in FTB

#### 3.7.1. Analysis of Bacterial Diversity and Composition

**Figure 4:**
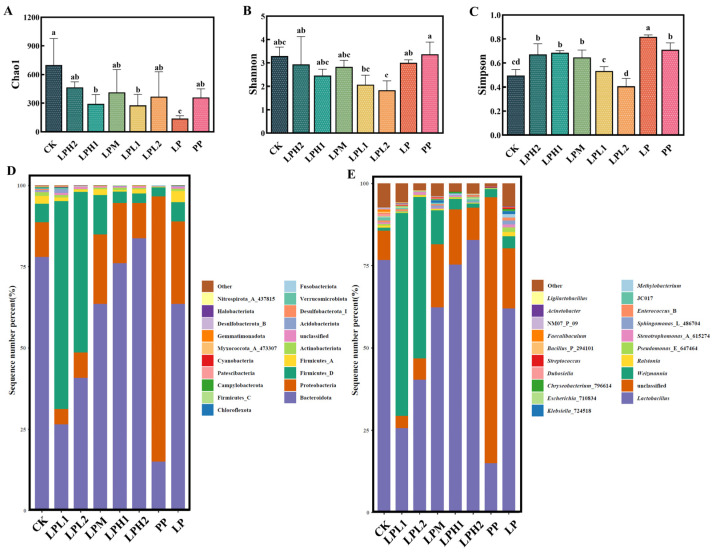
Bacterial diversity and composition. Chao1 index (**A**), Shannon index (**B**), Simpson index (**C**), phylum level (**D**), and genus level (**E**). CK: natural fermentation; LP: *L. plantarum* HYY-S10 monoculture; PP: *P. pastoris* monoculture; LPH2, LPH1, LPM, LPL1, LPL2: co-culture groups with *L. plantarum* HYY-S10 to *P. pastoris* inoculation ratios of 100:1, 10:1, 1:1, 1:10, and 1:100, respectively. Lowercase letters indicate significant differences among groups.

Microbial community analysis of FTB samples was performed through high-throughput sequencing of the V3-V4 hypervariable region of the 16S rDNA gene (Figure 4). The α-diversity indices, including Chao1 (richness estimator), Shannon (diversity index), and Simpson (dominance index), were employed to comprehensively evaluate the bacterial community diversity and structure [37]. As illustrated in Figure 4A-C, the results revealed significant variations in bacterial diversity patterns among different treatment groups, demonstrating that the introduction of varying concentrations of *L. plantarum* HYY-S10 substantially influenced both the taxonomic diversity and relative abundance of bacterial populations in the FTB ecosystem. Notably, the CK group exhibited the highest levels of bacterial richness among all experimental groups (*p* < 0.05) (Figure 4A), while both CK and PP groups maintained the highest bacterial diversity levels (*p* < 0.05) (Figure 4B). These findings align with the Simpson index results, where lower values (indicating higher diversity) were consistently observed in the CK group (*p* < 0.05) (Figure 4C). This observation suggests that spontaneous fermentation permits the development of a more phylogenetically diverse microbial consortium, likely comprising various indigenous bacteria present in the raw tea materials and fermentation environment. The reduced diversity observed in *L. plantarum*-treated groups may reflect the competitive dominance of the inoculated strain, which potentially suppresses the growth of other bacterial taxa through mechanisms such as nutrient competition, acid production, or bacteriocin secretion. These findings align with previous studies demonstrating that starter culture addition typically decreases microbial diversity while increasing fermentation process control and product consistency [38].

Microbial community profiling of the FTB samples revealed distinct taxonomic distributions at both phylum and genus levels (Figure 4D,E). the relative abundance of bacteria at the phylum level was highest for Bacteroidota (15.07–83.74%), followed by Proteobacteria (4.82–81.41%), and Firmicutes (3.08–64.99%) (Figure 4D). At the genus level, *Lactobacillus* (14.90–82.82%), unclassified taxa (3.70–80.86%), and *Weizmannia* (0.9–61.62%) represented the most abundant operational taxonomic units (Figure 4E). A dose-dependent relationship was observed between *L. plantarum* HYY-S10 inoculation levels and Lactobacillus genus abundance. This quantitative association suggests that the inoculated *L. plantarum* HYY-S10 effectively colonizes the fermentation matrix and competitively dominates the microbial community. The progressive increase in *Lactobacillus* abundance corresponding to higher inoculation doses indicates that microbial community structure in FTB fermentation can be precisely modulated through controlled starter culture application. Furthermore, the observed dominance of *Lactobacillus* at higher inoculation levels suggests its potential role as a keystone species in shaping the functional characteristics of the final fermented product.

#### 3.7.2. Analysis of Fungal Diversity and Composition

**Figure 5:**
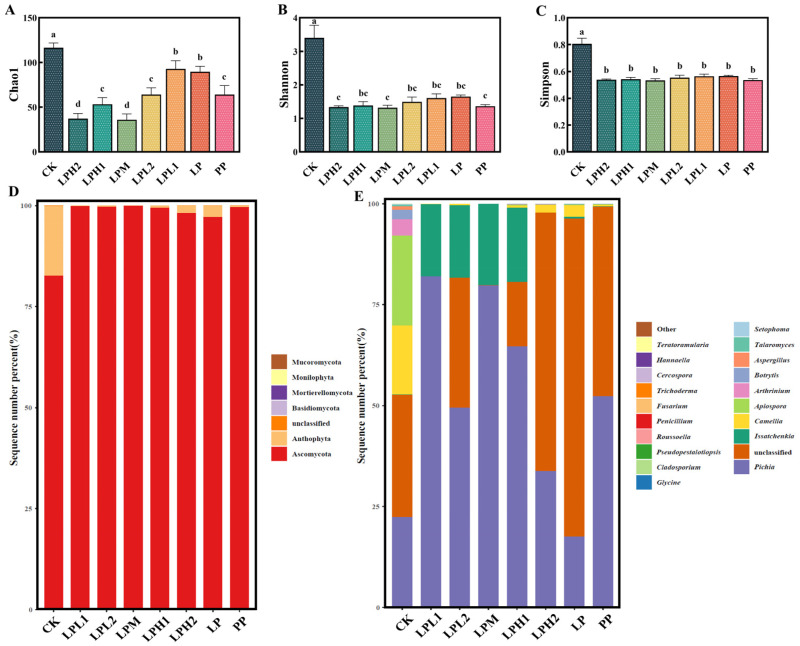
Fungal diversity and composition. Chao1 index (**A**), Shannon index (**B**), Simpson index (**C**), phylum level (**D**), and genus level (**E**). CK: natural fermentation; LP: *L. plantarum* HYY-S10 monoculture; PP: *P. pastoris* monoculture; LPH2, LPH1, LPM, LPL1, LPL2: co-culture groups with *L. plantarum* HYY-S10 to *P. pastoris* inoculation ratios of 100:1, 10:1, 1:1, 1:10, and 1:100, respectively. Lowercase letters indicate significant differences among groups.

Fungal community analysis through ITS gene sequencing revealed significant alterations in FTB microbiota (Figure 5). Analysis of fungal α-diversity revealed distinct patterns between experimental groups (Figure 5A–C). The species richness estimators (Chao1 indices) demonstrated that the LPH1, LPH2 and LPM groups were significantly reduced relative to the CK group (*p* < 0.05) (Figure 5A). This marked decrease in richness metrics suggests that the *L. plantarum* HYY-S10-*P. pastoris* co-culture system effectively suppressed fungal proliferation, likely through competitive exclusion or production of antifungal metabolites. In contrast, diversity indices (Shannon and Simpson) remained relatively stable across and minimal variation between groups all treatment groups except CK (Figure 5B,C), indicating that while co-fermentation quantitatively reduced fungal richness, it maintained comparable community evenness.

Fungal community composition analysis at the phylum level revealed a consistent dominance of Ascomycota throughout the fermentation process, representing 82.62–99.94% of the total fungal population (Figure 5D). This finding aligns with Huang et al.’s macro-genomics analysis of wine, where Ascomycota fungi constituted over 80% of the fungal population in wine [39]. Fungal community composition at the genus level exhibited substantial variation, with Pichia (17.54–81.98%), unclassified fungi (0.03–78.76%), and *Issatchenkia* (0.05–20.18%) emerging as the dominant taxa (Figure 5E). The numerically dominant *Pichia* population (17.54–81.98%) represents a functionally crucial component of the FTB ecosystem, enhances the glycerol and ethanol content of the wine system and imparts a unique fragrance [38]. *Issatchenkia* demonstrates resistance to multiple environmental stressors, maintaining activity in environments containing ethanol or organic acids [40]. Importantly, our results demonstrate that *L. plantarum* HYY-S10 inoculation dosage significantly altered both fungal community structure and taxonomic composition, with the system consistently selecting for *Pichia* and *Issatchenkia* as the predominant functional guilds.

### 3.8. Correlation of FTB Flavor Characteristic Metabolites and Active Microflora

**Figure 6:**
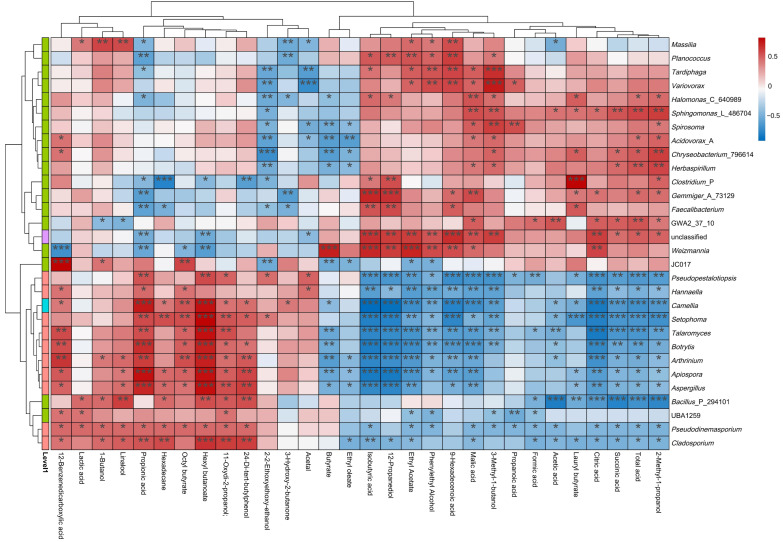
Correlation of flavor profile metabolites of tea beverage with active bacterial flora. The graphs display R-values in various colors, *p*-values (*) indicate *p*-values less than 0.05, the color bar on the left indicates the phylum categories to which the species belongs, and the caption on the right displays the color intervals of the various R-values. * indicates 0.01 ≤ *p *< 0.05, ** indicates 0.001 ≤ *p *< 0.01, *** indicates *p *< 0.00.

To elucidate the relationship between key microorganisms and the organic acids and flavor components in FTB, a correlation heat map analysis was conducted (Figure 6). Bacterial taxa including *Sphingomonas*, *Chryseobacterium*, *Weizmannia*, JC017, and *Bacillus* demonstrated extensive positive correlations with flavor compounds, suggesting their pivotal role as core functional microbiota. Notably, *Sphingomonas* exhibited significant associations (8 positive, 7 negative), while *Weizmannia* (8 positive, 4 negative) and *Bacillus* (7 positive, 7 negative) showed balanced metabolic interactions. In contrast, fungal genera (*Pseudopestalotiopsis*, *Camellia*, *Talaromyces*) primarily displayed negative correlations with flavor profiles, with *Pseudopestalotiopsis* (5 positive, 14 negative), Camellia (8 positive, 14 negative), and *Talaromyces* (5 positive, 14 negative) showing particularly inhibitory patterns. The observed microbial dichotomy suggests bacterial dominance in flavor metabolite production through two key mechanisms: preferential substrate utilization, exemplified by *L. plantarum*’s rapid glucose metabolism into lactic acid, creating a low-pH environment that selectively favors bacterial proliferation over fungi; and enzymatic biotransformation, where *L. plantarum*’s lactate dehydrogenase (LDH) generates pyruvate that *P. pastoris* subsequently converts via pyruvate decarboxylase (PDC) into characteristic flavor compounds like fruity acetoin and buttery diacetyl [40]. Notably, the *L. plantarum* HYY-S10-*P. pastoris* synergy enhances FTB quality through cross-feeding dynamics, where bacterial-derived lactic acid serves as a carbon source for yeast, promoting ethyl ester production via *P. pastoris*’s alcohol acetyltransferase (ATF1), while pH-mediated modulation (pH < 4.0) from *L. plantarum* HYY-S10 acidification simultaneously stabilizes yeast-derived flavor compounds and inhibits off-flavor microorganisms [41]. This multifaceted interaction creates an optimized biochemical environment for flavor development and microbial community stability.

## 4. Conclusions

The *L. plantarum* HYY-S10-*P. pastoris* co-fermentation system (1:1 ratio) significantly enhanced FTB quality, elevating antioxidant activity by 35% and key flavor compounds (e.g., 3-methyl-1-butanol, phenylethyl alcohol) by 2–3 fold compared to natural fermentation. High-throughput sequencing identified *Lactobacillus* (72.4% abundance) and *Pichia* (61.3%) as dominant taxa, with bacterial abundance strongly correlating (*p* < 0.05) with desirable flavor profiles. The demonstrated efficacy and microbial stability of this specific co-culture system provide a reliable and scalable model for industrial fermentation, offering a viable alternative to unpredictable natural fermentation processes. For practical application, future work should focus on pilot-scale validation to determine optimal aeration and agitation parameters for large-scale bioreactors. Furthermore, the adoption of quantitative GC-MS with authentic standards and multi-omics analyses in subsequent research will be crucial to precisely define critical quality control markers, thereby refining process control and ensuring the consistent production of high-quality FTB on an industrial scale.

## Figures and Tables

**Table 1:** Composition of FTB seasoning mixture.

Ingredient	LPH2	LPH1	LPM	LPL1	LPL2	LP	PP	CK
Glutinous rice (g)	50	50	50	50	50	50	50	50
Distilled water (mL)	400	400	400	400	400	400	400	400
Amylase (g)	0.65	0.65	0.65	0.65	0.65	0.65	0.65	0.65
Glucoamylase (g)	19	19	19	19	19	19	19	19
Green tea leaves (g)	8	8	8	8	8	8	8	8
*L. plantarum* (log CFU/mL)	8	7	6	5	4	6	-	-
*P. pastoris* (log CFU/mL)	6	6	6	6	6	-	6	-

CK: natural fermentation; LP: *L. plantarum* HYY-S10 monoculture; PP: *P. pastoris* monoculture; LPH2, LPH1, LPM, LPL1, LPL2: co-culture groups with *L. plantarum* HYY-S10 to *P. pastoris* inoculation ratios of 100:1, 10:1, 1:1, 1:10, and 1:100, respectively. -: no added.

## Data Availability

The original contributions presented in the study are included in the article/Supplementary Material, further inquiries can be directed to the corresponding author.

## Supplementary Materials

The following supporting information can be downloaded at https://www.mdpi.com/article/10.3390/foods14244251/s1, Table S1: Types and contents of main volatile compounds.
