# Supplementary material for: Co-Fermentation with Lactiplantibacillus plantarum and Pichia pastoris: A Novel Approach to Enhance Flavor and Quality of Fermented Tea Beverage
Source: Foods. 2025 Dec 10;14(24):4251. doi: 10.3390/foods14244251 (PMC12733218; doi:10.3390/foods14244251)
Supplement: Supplementary file 1 [file foods-14-04251-s001.zip › Supplementary material.pdf]

Table S1. Types and contents of main volatile compounds.

Number	Retention time (min)	Compounds	LPH2	LPH1	LPM	LPL1	LPL2	LP	PP	CK
			Relative content %							
		**Alcohols**								
1	12.3637	2-Methyl-1-propanol	1.05	1.08	1.36	1.16	1.08	0.76	1.08	0.75
2	15.8538	3-Methyl-1-butanol	9.28	9.02	8.39	9.56	8.20	7.65	10.72	2.97
3	17.5576	2-Heptanol, acetate	ND	ND	ND	ND	ND	ND	ND	0.27
4	19.0148	1-Butanol	ND	ND	ND	ND	ND	ND	ND	0.14
5	19.1911	2-Heptanol	ND	ND	ND	ND	ND	ND	ND	0.47
6	20.1253	1-Hexanol	ND	ND	ND	ND	ND	ND	ND	1.00
7	21.0007	Daucol	ND	ND	0.03	ND	ND	ND	ND	ND
8	21.9819	2-Octanol	ND	ND	ND	ND	ND	ND	ND	0.12
9	22.9162	Heptanol	ND	ND	ND	ND	ND	0.05	0.06	ND
10	24.6083	2-Nonanol	ND	ND	ND	ND	ND	ND	ND	0.72
11	25.3075	Linalool	0.41	0.19	0.18	0.26	0.21	0.19	0.38	0.73
12	25.5659	1-Octanol	ND	ND	ND	0.02	ND	ND	ND	ND
13	28.0397	Furfuryl alcohol	0.11	ND	0.09	ND	0.09	0.13	0.08	0.40
14	28.0456	3-Furanmethanol	ND	0.10	ND	0.11	ND	ND	ND	ND
15	29.0914	α-Terpineol	0.06	0.04	0.04	0.04	0.03	ND	0.04	ND
16	29.4733	3-Methylthiopropanol	0.10	0.09	0.14	0.09	0.08	0.16	0.13	ND
17	32.9986	Benzyl alcohol	ND	ND	ND	ND	ND	ND	ND	1.33
18	33.7743	Phenylethyl Alcohol	8.86	7.61	6.53	7.90	6.05	6.95	8.11	2.97
19	37.7579	Benzenemethanol,3,5-dimethyl-	ND	0.06	0.10	ND	ND	ND	ND	ND
20	37.758	3,4-Dimethylbenzyl alcohol;	0.12	ND	ND	ND	ND	ND	ND	ND

21	39.6146	1,1'-Oxydi-2-propanol	0.54	1.40	2.69	1.54	0.27	10.23	6.82	11.14
22	44.5324	1,2-Propanediol	31.14	5.13	46.22	3.72	4.72	0.06	27.70	1.20
23	50.3197	2-Mercapto-3-butanol	ND	ND	ND	0.38	ND	ND	ND	ND
		Total	51.67	24.72	65.77	24.78	20.73	26.18	55.12	24.21
		**Esters**								
24	4.1554	n-Propyl acetate	ND	ND	ND	0.32	ND	ND	ND	ND
25	4.1555	Ethyl 3-hydroxybutyrate	ND	ND	ND	ND	ND	0.37	0.31	ND
26	6.2707	Ethyl acetate	33.13	27.19	22.76	26.78	21.27	22.21	30.39	3.30
27	7.9688	Ethyl propionate	0.68	1.00	0.55	1.04	0.89	0.56	0.94	ND
28	12.9865	Isoamyl acetate	0.31	0.12	0.20	0.26	0.22	0.09	0.43	1.19
29	16.6	Ethyl caproate	0.04	ND	0.04	0.03	0.03	0.12	0.07	ND
30	17.1287	Isoamyl formate	ND	ND	ND	ND	ND	ND	ND	0.35
31	18.4038	3,7-Dimethyl-, acetate, (z)-6-octadien-1-ol	ND	ND	ND	ND	ND	ND	0.03	ND
32	18.4097	Ethyl butyrate	ND	ND	ND	ND	0.02	ND	ND	ND
33	22.4638	Ethyl caprylate	0.21	0.06	0.07	0.15	0.11	0.48	0.37	ND
34	24.4614	2,4-Hexadienoic acid, ethyl ester	ND	ND	0.12	ND	ND	ND	ND	ND
35	24.4732	Ethyl 2,4-hexadienate	0.17	ND	ND	0.14	ND	0.15	0.16	ND
36	24.5203	Methyl 2-furoate	ND	ND	ND	ND	0.09	ND	ND	ND
37	25.1313	Ethyl nonanoate	ND	ND	ND	ND	ND	0.08	ND	ND
38	26.6413	Methyl n-caprate	ND	ND	ND	0.06	0.04	ND	0.09	0.16
39	27.6637	Ethyl decanoate	0.08	0.06	0.06	0.04	0.06	0.40	0.11	ND
40	30.1197	Benzene	0.06	0.06	0.04	0.05	0.04	0.06	ND	ND
41	31.43	Methyl dodecanoate	ND	ND	ND	ND	0.03	ND	ND	ND
42	31.4417	Methyl 10-methylundecanoate	ND	ND	ND	ND	ND	ND	0.04	ND
43	31.7942	Phen isoamyl acetate	ND	ND	0.11	ND	0.10	0.14	0.14	ND

44	31.7943	Acetic acid, 2-chloro-,2-phenylethyl ester	0.15	ND	ND	ND	ND	ND	ND	ND
45	31.8059	Phenethyl isobutyrate	ND	ND	ND	ND	ND	ND	ND	0.17
46	32.3289	Ethyl laurate	0.09	0.07	0.06	0.07	0.05	0.19	0.11	ND
47	33.1925	2,2,4-Trimethyl-1,3-pentanediol diisobutyrate	ND	ND	0.04	ND	0.06	ND	ND	ND
48	33.1984	Butyl butyryllactate	ND	ND	ND	ND	ND	ND	ND	0.35
49	33.4687	9-Hexadecenoic acid,ethyl ester	1.50	0.87	1.05	1.05	0.76	0.39	1.09	0.43
50	35.6957	Isoamyl phenylacetate	0.02	ND	ND	ND	ND	ND	ND	ND
51	36.5828	Ethyl myristate	0.04	0.03	0.04	ND	ND	ND	0.08	0.22
52	37.3113	9-Hexadecenoicacid, ethyl ester	0.05	ND	ND	0.05	ND	0.05	0.12	ND
53	37.3231	Ethyl 9-tetradecenoate	ND	ND	0.04	ND	ND	ND	ND	ND
54	38.3102	Tricyclodecenyl Propionate	ND	ND	ND	0.04	ND	ND	0.02	ND
55	38.8097	Cyclopentadecanolide	ND	ND	ND	ND	0.03	ND	ND	ND
56	39.7615	Methyl hexadecanoate	ND	0.09	0.06	0.03	0.08	0.07	0.08	0.13
57	39.7674	Tridecanoic acid methyl ester	0.13	ND	ND	ND	ND	ND	ND	ND
58	39.7732	Methyl stearate	ND	ND	ND	ND	ND	ND	ND	0.31
59	40.2139	Butyl isovalerate	ND	ND	0.03	ND	ND	ND	ND	ND
60	40.2256	Ethyl 2-methylbutyrate	ND	ND	ND	ND	ND	ND	0.07	ND
61	40.4548	Ethyl palmitate	0.32	0.27	0.28	0.29	0.25	0.17	0.19	ND
62	41.8238	2-methyl-propanoic aci acetyl ester	0.10	0.46	ND	0.83	0.07	0.01	ND	ND
63	42.523	Lauryl butyrate	0.17	0.04	0.84	0.16	4.54	ND	0.33	ND
64	42.6994	(Z)-Ethyl heptadec-9-enoate	0.07	ND	ND	ND	ND	ND	ND	ND
65	43.2104	Hexyl butanoate	ND	0.12	0.03	0.29	0.08	2.48	1.79	4.29
66	44.4031	Ethyl oleate	0.49	0.31	0.35	0.24	0.37	0.42	ND	ND
67	44.4149	(E)-9-Octadecenoic acid ethyl ester	ND	ND	ND	ND	ND	ND	0.36	ND

68	44.5324	Octadecanoic acid,ethyl ester	ND	0.05	ND	ND	ND	ND	ND	ND
69	45.1905	Octadecadienoate	ND	0.69	ND	ND	ND	ND	ND	ND
70	45.1906	Linoleic acid ethyl ester	0.76	ND	0.57	ND	1.19	ND	ND	ND
71	45.4549	Diethyl phthalate	0.16	1.29	1.38	1.33	2.00	0.48	1.84	6.53
72	46.2892	Ethyl linolenate	0.33	ND	0.63	ND	0.96	ND	0.85	ND
73	47.3351	Ethyl-2-methyl-1,3-dioxolane -2-acetate	ND	ND	ND	ND	ND	ND	0.59	ND
74	47.7346	1,2-Propanediol, diacetate	ND	0.28	ND	ND	ND	ND	ND	ND
75	47.9344	Butyl octyl ester	ND	ND	ND	ND	ND	1.94	ND	ND
76	50.7547	α-Angelica lactone	ND	ND	ND	ND	ND	ND	0.10	ND
77	54.7794	Octyl butyrate	0.23	1.05	ND	0.33	1.97	0.20	0.69	2.18
		Total	39.29	34.10	29.36	33.57	35.31	31.07	41.42	19.62
		**Aldehydes**								
78	3.5386	Butyraldehyde	0.43	0.24	ND	ND	0.02	1.2	0.46	0.47
79	12.07	3-Methylbutanal	0.63	0.43	ND	ND	0.18	ND	ND	ND
80	21.0067	4-Hydroxy-3-methoxybenzaldehyd	ND	ND	ND	ND	0.03	ND	ND	ND
81	21.4295	n-Nonanal	ND	ND	ND	0.07	ND	ND	ND	ND
82	23.821	Dodecyl aldehyde	ND	ND	ND	ND	ND	ND	ND	0.15
83	25.0607	Benzaldehyde	ND	ND	ND	ND	ND	ND	ND	0.19
84	32.0057	3,4-Dimethylbenzaldehyde	ND	0.12	ND	ND	0.08	0.06	ND	ND
85	32.0058	4-Ethylbenzaldehyde	0.11	ND	ND	ND	ND	ND	ND	ND
86	32.0114	Benzaldehyde, 2-ethyl	ND	ND	ND	0.1	ND	ND	ND	ND
87	32.0115	2,6-Dimethylbenzaldehyde	ND	ND	ND	ND	ND	ND	ND	0.21
88	32.0175	3,5-Dimethylbenzaldehyde	ND	ND	0.07	ND	ND	ND	0.05	ND
89	38.8037	Citral diethyl acetal	ND	ND	ND	ND	ND	0.05	ND	ND
90	39.1739	2-Butyl-4-methyl-1,3-dioxolane	ND	0.05	ND	ND	ND	ND	ND	ND

91	40.02	2,6-Dihydromethyl-5-heptenal	0.61	0.03	0.19	ND	0.34	0.05	0.65	0.09
92	40.7604	Myrac aldehyde	ND	ND	ND	ND	0.04	ND	ND	ND
93	42.3292	Veratraldehyde	0.02	ND	ND	ND	ND	ND	ND	ND
94	48.8862	acetaldehyde PG acetal	ND	ND	ND	ND	ND	3.87	ND	ND
95	50.8486	3,7-Dimethyl-7-hydroxyoctanal	ND	0.04	ND	ND	ND	ND	ND	ND
96	52.0061	Acetal	0.11	0.26	1.03	ND	2.87	1.19	ND	0.98
97	56.407	Isobutanal	ND	ND	ND	ND	0.05	ND	ND	ND
		Total	1.91	1.17	1.29	0.17	3.61	6.42	1.16	2.09
		**Ketones**								
98	7.5104	3-Hydroxy-2-butanone	5.16	34.89	0.23	38.11	38.83	23.79	0.01	5.85
99	8.568	2-Pentanone;Methyl propyl ketone	ND	ND	ND	ND	ND	ND	ND	0.38
100	26.9468	Isophorone	0.07	0.06	0.06	ND	ND	ND	ND	0.24
101	37.7578	3'-Hydroxyacetophenone	ND	ND	ND	0.1	ND	ND	ND	ND
102	38.8037	2H-Pyran-4-ol, tetrahydro-3-pentyl-, acetate	ND	0.03	ND	ND	ND	ND	ND	ND
103	42.1058	g-Damascone	ND	ND	ND	ND	ND	0.04	ND	ND
104	47.2528	2-Tridecanone	ND	ND	ND	ND	ND	ND	0.29	ND
105	51.489	2,5,10-Trimethyl-2,5,9-cyclododecatrien-1-yl methyl ketone	ND	ND	0.04	ND	1.26	ND	ND	0.25
106	53.6513	5-Methyl-3-heptanone oxime	ND	0.02	0.03	ND	ND	ND	ND	ND
		Total	5.23	35.00	0.36	38.21	40.09	23.83	0.30	6.72
		**Acids**								
107	4.1555	Acetic acid	ND	ND	0.30	ND	ND	ND	ND	ND
108	18.3626	Hex-4-enoic acid	ND	ND	0.03	0.03	ND	ND	ND	ND
109	27.6811	8-Methylnonanoic acid, ethyl ester	ND	ND	ND	0.07	ND	ND	ND	ND

110	50.3198	Crotonic acid	ND	ND	0.36	ND	ND	ND	ND	ND
111	55.3611	Lactic acid	0.09	0.03	ND	1.33	ND	0.15	0.10	6.39
		Total	0.09	0.03	0.69	1.43	0.00	0.15	0.10	6.39
		**Alkanes**								
112	3.8147	n-Heptacosane	ND	ND	ND	ND	ND	ND	0.06	ND
113	17.1111	Pentadecane	ND	ND	ND	ND	0.04	ND	ND	ND
114	18.6975	Octadecane	ND	ND	ND	ND	ND	0.04	0.07	ND
115	18.7269	n-Tridecane	ND	0.04	ND	ND	ND	ND	ND	ND
116	18.7444	Eicosane	ND	ND	ND	0.04	ND	ND	ND	ND
117	26.7941	Hexadecane	0.07	0.09	0.06	0.09	0.05	0.08	0.07	0.33
118	27.7694	n-Tetracosane	ND	ND	ND	ND	ND	ND	0.16	0.30
119	28.4979	2,6,10,14-tetramethylpentadecane	ND	ND	ND	ND	ND	ND	ND	0.26
120	28.9269	Dotriacontane	ND	ND	ND	ND	ND	ND	ND	0.27
121	29.1678	Heptadecane	0.07	0.04	0.03	0.04	ND	0.04	0.06	0.75
122	40.2021	n-Pentadecane	ND	ND	ND	ND	ND	0.04	ND	ND
123	45.3667	1,4,7,10,13,16-Hexaoxacyclooctadecane	ND	ND	ND	ND	ND	ND	ND	4.19
124	53.2576	Boisambrene forte	ND	ND	ND	ND	ND	2.11	0.86	ND
		Total	0.14	0.17	0.09	0.17	0.09	2.31	1.28	6.10
		**Olefin**								
125	15.3191	D-Limonene	ND	ND	ND	0.04	ND	0.03	0.06	0.26
126	29.0797	γ-Terpinene	ND	ND	ND	ND	ND	0.04	ND	ND
127	30.1549	α-Cuprenene	ND	ND	ND	ND	ND	ND	ND	0.24
128	31.1596	α-cedrene	ND	ND	ND	ND	ND	ND	ND	0.19
129	37.7579	α-terpinolene	ND	ND	ND	ND	ND	0.05	ND	ND
130	38.8037	1-Dodecene	ND	ND	0.02	ND	ND	ND	ND	ND

131	38.9682	γ-selinene	ND	ND	ND	ND	ND	0.01	ND	ND
		Total	0	0	0.02	0.04	0	0.13	0.06	0.69
		**Others**								
132	3.8088	2-Ethyl-oxetane	ND	ND	ND	ND	ND	ND	ND	1.19
133	30.1314	Benzothiazole	ND	ND	ND	ND	ND	ND	0.10	ND
134	31.8	Acetamide,N-(2-phenylethyl)-	ND	0.13	ND	0.15	ND	ND	ND	ND
135	36.242	Phenol	ND	ND	ND	ND	ND	ND	ND	0.69
136	37.3291	Sandela I	ND	ND	ND	ND	0.03	ND	ND	ND
137	40.7486	2,4-Dimethylthiazole	ND	ND	ND	ND	ND	0.01	ND	ND
138	40.7662	Diallyl trisulfide	ND	ND	0.03	ND	ND	ND	ND	ND
139	41.1951	2,4-Di-tert-butylphenol	0.14	0.18	0.11	0.24	0.12	0.14	0.23	1.08
140	43.8274	4-Pyridinemethanamine	ND	ND	ND	ND	ND	ND	0.12	ND
141	45.1905	Diethylene glycol monoethyl ether	1.52	4.44	2.07	1.12	0.04	9.79	ND	4.14
142	47.6523	Octaethylene glycol monododecyl ether	ND	ND	ND	ND	ND	ND	ND	21.76
143	49.5619	Indole	ND	ND	ND	0.13	ND	ND	ND	3.81
144	49.9967	p-Hydroxyallylbenzene	ND	ND	0.21	ND	ND	ND	ND	ND
145	50.3316	Dipropylene glycol monomethyl ether	ND	ND	ND	ND	ND	ND	ND	1.50
		Total	1.66	4.75	2.42	1.64	0.19	9.94	0.45	34.17

"ND": not detected. CK refers to the natural fermentation; LP refers to *L. plantarum*; PP refers to the *P. pastoris*; LPH2 refers to *L. plantarum* and *P. pastoris* inoculation ratio of 100:1; LPH1 refers to *L. plantarum* and *P. pastoris* inoculation ratio of 10:1; LPM refers to *L. plantarum* and *P. pastoris* inoculation ratio of 1:1; LPL1 refers to *L. plantarum* and *P. pastoris* inoculation ratio of 1:10; LPL2 refers to *L. plantarum* and *P. pastoris* inoculation ratio of 1:100.
